# Effect of Sociodemographic Factors, Concomitant Disease States, and Measures Performed in the Emergency Department on Patient Disability in Ischemic Stroke: Retrospective Study from Lebanon

**DOI:** 10.1155/2021/5551558

**Published:** 2021-05-27

**Authors:** Diana Malaeb, Souheil Hallit, Hiba Al Harfany, Sara Mansour, Frederic Faugeras, Pascale Salameh, Hassan Hosseini

**Affiliations:** ^1^School of Pharmacy, Lebanese International University, Beirut, Lebanon; ^2^Life Sciences and Health Department, Paris-Est Créteil University, Paris, France; ^3^Faculty of Medicine and Medical Sciences, Holy Spirit University of Kaslik (USEK), Jounieh, Lebanon; ^4^INSPECT-LB: Institut National de Santé Publique, Epidémiologie Clinique et Toxicologie, Beirut, Lebanon; ^5^AP-HP, Hôpital Henri Mondor-Albert Chenevier, Department of Neurology, Créteil, France; ^6^Department of Cognitive Sciences, École Normale Supérieure, PSL University, Paris, France; ^7^Medical School, University of Paris XII, Créteil, France; ^8^Mondor Institute for Biomedical Research, INSERM U955, Créteil, France; ^9^Faculty of Pharmacy, Lebanese University, Hadat, Lebanon; ^10^University of Nicosia Medical School, Nicosia, Cyprus; ^11^Stroke Unit, Service de Neurologie, CHU Henri Mondor - 94010, Créteil Cedex, France; ^12^UPE-C, Université Paris-Est Créteil, Faculté de Santé, Paris, France; ^13^INSERM U955-E01, IMRB, Créteil, France

## Abstract

**Background:**

Stroke is a leading cause of death and disability in developed countries. The major factor affecting long-term survival other than age is the disability severity caused by stroke. The modified Rankin Scale (mRS) is a global functional endpoint measurement used in acute stroke to evaluate the degree of disability or dependence in daily life activities. The objective of this study was to assess the effects of sociodemographic factors, concomitant disease states, and some measures performed in the emergency department (ED) on patients' disability.

**Methods:**

We conducted a retrospective study on ischemic stroke patients admitted to Intensive Care Unit of three Lebanese university hospitals between June and December 2016. Patients were excluded if they had been discharged from ED without hospital admission or if mRS was not performed. The mRS was further subdivided into two categories considered as “good prognosis” (0-2 or 0-3) and “poor prognosis” (>2 or > 3).

**Results:**

204 patients were included in the study with mean age of 65.4 ± 11.9 years, hypertension was the most previous concomitant past medical disease (77.1%), and 27.1% of these patients had previous history of stroke. No significant differences were found in both mRS categories for all sociodemographic factors, and past medical history except that arrhythmia was significantly more common in the higher mRS categories > 2 and > 3. Based on multivariable analysis, there was a trend for previous intake of calcium channel blocker to be associated with lower mRS at admission (beta -0.586). However, intracranial arterial stenosis, ED blood glucose > 180 mg/dL, and performing brain imaging above 20 minutes after patient presentation to ED were significantly associated with higher mRS scores at discharge with an ORa and (confidence interval) of 2.986 (0.814, 10.962), 3.301 (1.072, 1.261), and 1.138 (1.071, 9.080), respectively.

**Conclusion:**

mRS is affected by previous disease states, prescribed medications, and acute measures performed in ED. It is also influenced by intracranial arterial stenosis etiology, which is associated with worse outcome.

## 1. Introduction

Stroke is the third leading cause of mortality after heart diseases and cancer and a principal cause of severe long-term disability in adults [1]. The majority of strokes (80–85%) are of ischemic origin [2, 3].

In the last decade, several outcome measures were developed to assess the degree of poststroke disability, such as modified Rankin Scale (mRS), Barthel Index (BI), health-related quality of life (EQ5D-3L) [4]. However, mRS is a global functional endpoint measurement and the most frequently used index in acute stroke to evaluate the degree of disability or dependence in daily life activities following stroke and thereby easily communicate effects of treatments to physicians and patients [4, 5]. This scale is also a standard element in clinical practice and an outcome measure that must be used in all stroke survivors according to the Get with the Guidelines [6]. The mRS is an ordinal disability score which categorizes patients among 7 levels ranging from 0 “no disability” to 6 “death” [7].

In clinical practice, interpretation of mRS is challenging since it is a nonlinear scale; thus, dichotomization of outcomes has been performed in research studies to ensure consistent scoring, minimization of both subjective judgment, and variability in score assignment [8–11]. Indeed, in clinical trials, mRS is often further subdivided into two categories according to a cut-off value of either 2 or 3 defining good prognosis as mRS < 3 or < 4 and poor prognosis otherwise [12]. However, in current practice, even if patients with mRS of 3 are grouped with patients with mRS of 4-6 with the basic assumption that score of 3 is more similar to 4 than to 2 in terms of clinical outcomes, it raises concerns regarding the validity of this dichotomization given that mRS of 3 is typically considered of good clinical outcome and that patients with mRS 2 or 3 share a similar 7-year survival [13, 14]. The optimal and most objective cut-off value for mRS dichotomization is debatable as it depends on stroke severity, lacks the incorporation of the entire possible range of outcomes across the mRS, and raises difficulty as regards the interpretation of borderline score values of 2 and 3.

Several investigators performed poststroke outcome measurement following the entire ordinal distribution values of mRS, as this strategy is more powerful than the dichotomization approach, especially because it takes into consideration treatment effect that might occur over the entire range of mRS [15]. Result performance using the entire distribution of mRS values has greater statistical power as well than the dichotomized approach, mainly when treatment benefit is a continuous process [16].

Although mRS is widely applied for evaluating stroke patient outcomes, it has several pitfalls when used to measure poststroke disability. An extensive literature documents the negative effect of patient comorbidities including cardiovascular disease, diabetes, arthritis, surgery, and socioeconomic factors on physical functioning, cognitive abilities, and overall health status so that these factors may have detrimental effect on the mRS [17–22]. This is extremely important as comorbidities are common in stroke patients, and stroke incidence in low socioeconomic populations is especially high [23]. It is essential for clinicians to take into account these various attributes and avoid misapplication and misinterpretation of mRS. Hence, the objective of this study was to compare the effects of sociodemographic factors, concomitant disease states, and some measures performed in the ED on mRS.

## 2. Methods

### 2.1. Study Design

This was a retrospective observational study in which hospital records of all patients admitted to the Intensive Care Unit (ICU) of three Lebanese university hospitals between June and December 2016 were reviewed for inclusion. The list of hospitals, provided by the Lebanese Ministry of Public Health, was used to randomly select the centers. All adult patients presented to the emergency department with the diagnosis of ischemic stroke during the study period and who were subsequently admitted to the ICU were included in the study. No attempt was made to verify accuracy of the physician's diagnosis, because the aim of this study was to assess the impact of sociodemographic factors and medical history on mRS prognosis. Patients were excluded if the patient had been diagnosed with transient ischemic attack, had been discharged from the ED without hospital admission, or if the mRS was not available. Eligible participants were randomly selected from each center using the list generated from the hospital administrator for all patients diagnosed with ischemic stroke from the medical record department for possible enrollment in the study.

### 2.2. Data Collection

A medical record review was performed on site by the principal investigator, through a standardized data collection sheet. We collected baseline information including patient demographics, vascular risk factors, stroke severity (mRS at admission and upon discharge), and acute stroke management. mRS was documented by the attending physician on all patients' medical charts both upon presentation to the emergency department and upon discharge.

Vascular risk factors included hypertension, dyslipidemia, atrial fibrillation, previous stroke, smoking history, alcohol consumption, marital status, and body mass index (BMI) at admission which was categorized into underweight, normal, overweight, and obese [24]. Hypertension was defined as systolic blood pressure ≥ 140 mmHg or diastolic blood pressure ≥ 90 mmHg, any use of antihypertensive drug, or self-reported history of hypertension. We also included detailed assessment of antihypertensive medications including dose, frequency of administration, and therapeutic class [25]. Diabetes mellitus was defined as fasting glucose level ≥ 7.0 mmol/L, nonfasting glucose concentration ≥ 11.1 mmol/L, use of any glucose-lowering drugs, or self-reported history of diabetes [26]. Dyslipidemia was defined as serum triglyceride ≥ 1.7 mmol/L, low-density lipoprotein cholesterol ≥ 2.6 mmol/L, high-density lipoprotein cholesterol ≤ 1.0 mmol/L, use of any lipid-lowering drugs, or self-reported history of dyslipidemia [27]. Atrial fibrillation was defined as history of atrial fibrillation confirmed by at least one electrocardiogram or presence of arrhythmia during hospitalization. The treatment of atrial fibrillation defined by the use of anticoagulation agents or antiplatelet drugs during hospitalization and after discharge was also considered [28]. We followed the diagnosis of ischemic stroke set by the physician on the medical chart which is “an episode of neurological dysfunction caused by focal cerebral, spinal, or retinal infarction based on pathological, imaging, or other objective evidence of ischemic injury in a defined vascular distribution.” Etiologic subtypes of ischemic stroke were classified into cardioembolic, intracranial arterial stenosis, or other origins [29].

In addition, neurological assessment was documented through mRS. We then subdivided the mRS into two categories as “good prognosis” (either 0-2 or 0-3) and poor prognosis (either >2 or> 3) [12].

Data collection also gathered information about all measures done in the ED including brain imaging performance delay (within 20 minutes versus >20 minutes from hospital presentation) and administration of antiplatelet and antihypertensive medications.

### 2.3. Outcomes

The primary outcome was to assess the impact of different factors including prestroke sociodemographic factors and medication prescriptions along with etiologic subtypes of stroke on mRS scores both at hospital admission and discharge. The secondary outcome was to assess the impact of different measures performed in the hospital like brain imaging delay, glucose level, and antithrombotic therapy in the ED on mRS at discharge.

### 2.4. Statistical Analysis

The questionnaires were coded, and the collected data were introduced using the Statistical Package for Social Sciences (SPSS) software, version 23.0 by an independent person who was unaware of the objectives of the study. All continuous variables were presented as mean and standard deviation (SD), and categorical variables were presented as percentages. Correlations between sociodemographic factors, medical history, and mRS categories were determined by the Pearson chi-square test or Fisher's exact test when Pearson chi-square test could not be applied. Paired sample *t*-test was used to assess mean difference between mRS values at hospital admission and discharge.

Multiple logistic regression models were used to assess association between sociodemographic factors, medical history that showed a *P* < 0.2 in the bivariate analysis and mRS both at hospital admission and discharge (taken as the dependent variable). Potential confounders may be eliminated only if *P* > 0.2, in order to protect against residual confounding. We also performed a linear regression taking both mRS at hospital admission and discharge as the dependent variable and all other variables as independent. We ensured model adequacy by the use of the Hosmer Lemeshow test which is done for the logistic variables (mRS categories) to calculate if the observed event rates match the expected event rates. A *P* < 0.05 was considered as statistically significant.

## 3. Results

### 3.1. Sociodemographic Characteristics of Participants

From a total number of 400 patients screened for possible inclusion in the study, 204 subjects had documented mRS values at hospital admission and discharge and were enrolled in the study. Among the 204 participants, 132 (64.7%) were males. Mean age was 65.4 ± 11.9 years. Concerning BMI categories, 12 (29.3%) patients were normal, 17 (41.4%) overweight, and 12 (29.3%) obese. Almost half of the participants were smokers (48.5%), while the majority was nonalcoholic (64.7%), 75.1% were married, and 52.9% lived in Beirut district. Only 0.5% of the participants were physically active. As regards past medical history, hypertension was present in 77.1%; previous stroke or TIA prevalence was 27.1%. Atrial fibrillation, hyperlipidemia, and coronary artery bypass graft (CABG) were, respectively, found in 12.5%, 19.8%, and 4.2% of the participants. Regarding past medication history, 5.7% of the patients were on oral vitamin K antagonists, 0.5% on Low Molecular weight Heparin (LMWH), 38.8% on aspirin, 19.2% on clopidogrel, and 36.1% on lipid lowering therapy. Among patients with hypertension, 55.4% were on beta blockers, 34% were on calcium channel blockers (CCBs), and 25.3% were on dual antihypertensive drugs. The mean mRS scores at baseline and upon discharge were, respectively, 4.24 ± 0.80 and 2.51 ± 1.75 (see Table 1).

### 3.2. Demographic Data, Past Medication History, and Distribution Relative to mRS Categories at Discharge

An association between past medical history and mRS was found significant for arrhythmia. Indeed, arrhythmia was significantly associated with a poor prognosis (*P* = 0.003 for mRS > 2 versus 0-2 and 0.017 for mRS > 3 versus 0-3). Conversely, calcium channel blocker intake was significantly associated with a good prognosis but only in the comparison mRS 0-3 versus >3 (*P* value = 0.046) (see Table 2). No other association was found in our study.

### 3.3. Stroke Etiology and Diagnostic Factors

An association was found for cardioembolic etiology and poor prognosis but only for the mRS 0-2 versus >2 comparison (*P* value = 0.008). Lower mRS was found in the atherosclerotic or undetermined etiologies compared to the cardioembolic one (see Table 3).

### 3.4. Medication Administration in Hospital Relative to mRS Categories

An association was found between clopidogrel administration within 24-48 hours and good prognosis but only in the mRS 0-2 versus >2 comparison (*P* value = 0.047). No other significant association was found (see Table 4).

### 3.5. Regression Model for mRS upon Hospital Admission

We found a trend for intracranial arterial stenosis etiology to be associated with higher mRS values upon hospital admission. Conversely, previous intake of oral anticoagulant or calcium channel blockers was associated with lower mRS at admission. These associations were true both in the two and three category comparisons, but they were not significant (see Table 5). The results of the stepwise linear regression, taking the mRS upon admission as the dependent variable, showed that previous intake of calcium channel blocker was significantly associated with a decrease in the mRS score (beta = −0.568), whereas previous history of arrhythmia, diabetes, and percutaneous coronary intervention (PCI) with stent placement was associated with a nonsignificant increase of this score (beta = 0.451, 0.404, and 0.565, respectively) (see Table 6).

### 3.6. Logistic Regression Model for mRS upon Discharge

In logistic regression, the mean score difference in mRS between hospital admission and discharge was 1.73 ± 1.41 (*P* value <0.001).

For the two category mRS logistic regressions, patients having intracranial arterial stenosis were significantly associated with higher mRS values (OR = 4.14) with a confidence interval between 1.103 and 15.574. Also, a delay in the performance of brain imaging defined as at least 20 minutes after patient presentation to the ED was significantly associated with higher mRS values with an OR of 1.138 with a confidence interval between 1.071 and 9.080. In addition, prolonged hospital stay was significantly associated with higher mRS with a confidence interval of 1.104 and 1.173 and OR of 1.105. For the three category mRS logistic regression, patients having elevation in blood glucose levels defined by more than 180 mg/dL in the ED (OR = 3.301) were significantly associated with higher mRS values with a confidence interval between 1.072 and 1.261 (see Table 7). In addition, prolonged hospital stay was significantly associated with higher mRS in the three category as in the two category with a confidence interval of 1.014 and 1.109 and OR of 1.061.

### 3.7. Multiple Regression for Continuous mRS

In linear regression taking the mRS upon hospital discharge as the dependent variable and the mRS upon hospital admission as independent variable, we found a significant association between an increase in mRS upon hospital admission and the score upon hospital discharge with a beta of 0.581 and *P* value <0.001 (see Table 8).

## 4. Discussion

To the best of our knowledge, this is the first study that evaluates the association between sociodemographic factors, concomitant disease states, and some blood and imaging measures performed in the emergency department on the modified Rankin Scale score in a sample of Lebanese stroke patients. The results showed that having intracranial arterial stenosis, performing brain imaging above 20 minutes after arrival to the ED, and having serum blood glucose above 180 mg/dL were associated with a higher mRS score at discharge (worse prognosis).

In this paper, results were presented based on the two category and three category mRS score despite limitations of this approach already mentioned in Introduction. Note that our results were different according to the dichotomization threshold, which leads us to recommend using the continuous variable without any dichotomization [29–31].

Our results demonstrate that mRS at hospital admission is a potential indicator for mRS at hospital discharge. Indeed, we found a positive association between mRS at hospital admission and mRS at hospital discharge so that patients with higher mRS at admission were more likely to have higher mRS at discharge. These findings are broadly consistent with those of Kwok et al. who also found that prestroke mRS was a predictor of poststroke outcome and that poor prestroke mRS was associated with poor prognosis reflected by mortality and length of hospital stay [30]. In other words, patients with major disability at admission are unlikely to have a major change in functional status, regardless of hospital events. However, note that our study did not assess mortality and poststroke complications.

From a therapeutic point of view, this study demonstrates a trend for calcium channel blockers to be significantly associated with a lower mRS scores upon admission. This could be explained in part by the fact that CCBs can generate stronger antihypertensive effect through dilatation of blood vessels, lowering of blood pressure thereby exerting a protective effect [31]. Furthermore, it was documented that CCB also minimizes central wall changes that protects against stroke recurrence which is associated with good prognosis manifested with lower mRS scores [32].

Our results showed that intracranial arterial stenosis was associated with a higher mRS score (worse prognosis) both at admission and discharge, in line with previous findings which reported that neurologic deterioration was more common in intracranial arterial stenosis [33, 34]. Our results support previous findings that showed that large-artery atherosclerotic stroke had higher severity than small-vessel disease stroke and are associated with early recurrent stroke and worse prognosis [35–38].

Our results showed that a delay in performance of brain imaging defined as more than 20 minutes after patient presentation to the ED was significantly associated with a higher mRS score [39, 40]. The importance of early imaging in stroke extends far beyond its vital role in diagnosis [41]. Indeed, early brain imaging, in particular with magnetic resonance imaging (MRI), is fundamental not only for diagnosis but also for determining treatment strategies and evaluating stroke mechanisms [42–44].

One of the underlying reasons for this association is that patients with less severe stroke symptoms were more likely than those with more severe symptoms to experience a neuroimaging delay [36].

Another explanation is that patients who benefit of an earlier imagery can be treated early with a better prognosis [36–39].

In our study, having a high blood glucose (>180 mg/dL) when arriving to the ED was associated with bad prognostic score (mRS). Those results support the findings of a previous study that showed a higher risk for intracranial atherosclerotic disease found in patients with diabetes mellitus and metabolic syndrome due to catecholamine-mediated stress response leading to a more severe stroke [40]. The acute elevation in blood glucose levels during ischemic stroke is principally due to the activation of the hypothalamus pituitary adrenal axis and the sympathetic nervous system induced by stress leading to the release of cortisol and catecholamine which increase glucose levels [41]. Several studies reported worse outcomes in patients with acute ischemic stroke who exhibit stress hyperglycemia, and hyperglycemia on admission has been shown to be an independent predictor for intracerebral hemorrhage and for mortality after acute stroke [42–44]. The underlying reason for the link between hyperglycemia and higher mRS that accompanies acute stroke can be explained by the exacerbation of postischemic brain injury, amplification of cerebral edema, and transformation into hemorrhagic stroke. Even acute slight elevation in blood glucose is associated with a longer hospitalization, higher mortality rate, and increased infarct volume evaluated by MRI [45–48].

As for length of hospitalization and assessment of stroke functional outcome through the mRS, there was linear relationship between those two variables. The result is consistent with another study done in Saudi Arabia that documented that longer length of hospitalization was associated with bad functional outcomes upon discharge [48]. The underlying reason behind the relation between prolonged length of hospital stay and worse outcomes is that patients with comorbid diseases have bad outcomes that mandate longer term therapy, intensive rehabilitation, and closer monitoring to minimize irreversible damage.

The study showed that patients with previous history of arrhythmia had significantly higher risk of worse prognosis defined as higher mRS scores consistent with previous literature [49]. The proposed mechanism that delineates clearly the association between arrhythmia and worse outcome is the autonomic imbalance modulated by direct injury to neurogenic structures which is triggered by catecholamine surge leading to myocardial damage [50].

### 4.1. Limitations

The present study was limited by its retrospective design that precludes the ability to control for all potential confounders as patient demographics, clinical characteristics, and other factors that can affect the mRS scores as medications and diseases. Selection bias might play a role in the results since some patient's data were missing explaining the number of patients that were screened but not enrolled in the study. We used in this study, the mRS although National Institutes of Health Stroke Scale (NIHSS) is a widely used tool built to assess the cognitive effects of a stroke providing a quantitative measure of stroke-related neurologic deficit, yet the medical charts included mainly the mRS rather than the NIHSS. Since the study was dependent mainly on the medical charts, some data was missing as evaluation of patient status after discharge, prestroke functional status, and time from stroke onset to hospital admission that can lead to residual cofounding. The study is also limited by the use of a data set from certain Lebanese geographic areas which may limit the ability to generalize the results to a more heterogeneous population. Also, we did not use correction for multiple comparisons. In addition, this study had a major limitation as we did not assess mortality and poststroke complications although it is important to know how many patients were diagnosed with stroke at admission and died later in hospitalization and what was their modified ranking scale at admission. Furthermore, this study did not assess patients who had been treated through mechanical thrombectomy as those patients are treated in specialized units. Despite its limitations, this study does provide the basis for future studies to assess the influential factors on mRS and delineate the detrimental effects of blood glucose, stroke etiology, and brain imaging delay. It also provides support for the conduct of prospective study on the variables that affect mRS. Finally, the dissimilar results that we obtained according to the definition of the mRS (dichotomous versus continuous) also need to be taken into account in future studies.

## 5. Conclusion

In conclusion, acute stress hyperglycemia, intracranial arterial stenosis, and delayed brain imaging are associated with worse outcomes in acute ischemic stroke. The mRS, a common measure in acute stroke, is typically used to depict patient functional outcome and predict mortality. Thus, mRS is highly influenced by stroke severity, acute management, and concomitant disease states.

### 5.1. Implications for Practice

- Raise the need to thoroughly fill in the medical charts of ischemic stroke patients to minimize missing critical values that affect the prognosis
- Promote the documentation of NIHSS for all stroke patients upon admission rather than mRS
- Measure diseases that worsen stroke prognosis as blood glucose, temperature, and blood pressure

## Figures and Tables

**Table 1:** Sociodemographic characteristics of the study population.

Variable	*N* (%)
Mean age	65.40 years
Gender	
Male	132 (64.7)
Female	72 (35.3)
BMI	
Normal	12 (29.3)
Overweight	17 (41.4)
Obese	12 (29.3)
Marital status	
Single	39 (21.1)
Married	139 (75.1)
Divorced	7 (3.8)
Residence area	
Beirut	108 (60.3)
Mount Lebanon	33 (18.4)
Bekaa	5 (2.8)
North Lebanon	2 (1.1)
South Lebanon	31 (17.3)
Cigarette smoker	
Yes	99 (48.5)
Alcohol consumption	
Yes	21 (11.4)
Past medical history	
Hypertension	74 (77.1)
Stroke or TIA	26 (27.1)
Atrial fibrillation	12 (12.5)
Hyperlipidemia	19 (19.8)
Baseline mRS upon admission mean	1 interquartile range (2, 6)
Baseline mRS upon hospital discharge mean	2 interquartile range (0, 6)

**Table 2:** Effects of demographics and past medical history on mRS at discharge.

		Two category mRS		*P* value	Three category mRS		*P* value	
		0-2	>2		0-3	>3		
Gender	Male	31 (50.0%)	13 (36.1%)	0.183	34 (45.3%)	10 (43.5%)	0.867	
	Female	31 (50.0%)	23 (63.9%)		41 (54.7%)	13 (56.5%)		
Marital status	Married	21 (91.3%)	14 (82.4%)	0.397	28 (90.3%)	7 (77.8%)	0.316	
	Divorced/widowed	2 (8.7%)	3 (17.6%)		3 (9.7%)	2 (22.2%)		
Hypertension	Yes	45 (72.6%)	28 (77.8%)	0.61	57 (76%)	16 (69.6%)	0.435	
	No	15 (24.2%)	6 (16.7%)		16 (21.3%)	5 (21.7%)		
	Not documented	2 (3.2%)	2 (5.6%)		2 (2.7%)	2 (8.7%)		
Arrhythmia	Yes	14 (22.6%)	20 (55.6%)	0.003	21 (28.0%)	13 (56.5%)	0.017	
	No	45 (72.6%)	14 (38.9%)		51 (68%)	8 (34.8%)		
	Not documented	3 (4.8%)	2 (5.6%)		3 (4%)	2 (8.7%)		
Congestive heart failure	Yes	8 (12.9%)	7 (19.4%)	0.559	11 (14.7%)	4 (17.4%)	0.402	
	No	52 (83.9%)	27 (75%)		62 (82.7%)	17 (73.9%)		
	Not documented	2 (3.2%)	2 (5.6%)		2 (2.7%)	2 (8.7%)		
Angina pectoris	Yes	16 (25.8%)	10 (27.8%)	0.874	20 (26.7%)	6 (26.1%)	0.996	
	No	43 (69.4%)	25 (69.4%)		52 (69.3%)	16 (69.6%)		
	Not documented	3 (4.8%)	1 (2.8%)		3 (4%)	1 (4.3%)		
Percutaneous coronary intervention without stent	Yes	2 (3.2%)	2 (5.6%)	0.763	3 (4%)	1 (4.3%)	0.994	
	No	57 (91.9%)	33 (91.7%)		69 (92%)	21 (91.3%)		
	Not documented	3 (4.8%)	1 (2.8%)		3 (4%)	1 (4.3%)		
Coronary artery bypass graft	Yes	3 (4.8%)	3 (8.3%)	0.706	4 (5.3%)	2 (8.7%)	0.836	
	No	56 (90.3%)	32 (88.9%)		68 (90.7%)	20 (87%)		
	Not documented	3 (4.8%)	1 (2.8%)		3 (4%)	1 (4.3%)		
Peripheral arterial disease	Yes	3 (4.8%)	1 (2.8%)	0.839	3 (4%)	1 (4.3%)	0.943	
	No	55 (88.7%)	32 (88.9%)		67 (89.3%)	20 (87%)		
	Not documented	4 (6.5%)	3 (8.3%)		5 (6.7%)	2 (8.7%)		
Vitamin K antagonist	Yes	8 (12.9%)	10 (27.8%)	0.069	11 (14.7%)	7 (30.4%)	0.186	
	No	46 (74.2%)	25 (69.4%)		56 (74.7%)	15 (65.2%)		
	Not documented	8 (12.9%)	1 (2.8%)		8 (10.7%)	1 (4.3%)		
Antiplatelet therapy aspirin	Yes	24 (38.7%)	17 (47.2%)	0.568	33 (44%)	8 (34.8%)	0.73	
	No	29 (46.8%)	16 (44.4%)		33 (44%)	12 (52.2%)		
	Not documented	9 (14.5%)	3 (8.3%)		9 (12.0%)	3 (13%)		
Lipid lowering therapy	Yes	20 (32.3%)	8 (22.2%)	0.282	25 (33.3%)	3 (13%)	0.151	
	No	35 (56.5%)	26 (72.2%)		43 (57.3%)	18 (78.3%)		
	Not documented	7 (11.3%)	2 (5.6%)		7 (9.3%)	2 (8.7%)		
Beta blockers	Yes	3 (5.6%)	1 (3%)	0.731	3 (4.5%)	1 (5%)	0.534	
	No	48 (88.9%)	31 (93.9%)		60 (89.5%)	19 (95%)		
	Not documented	3 (5.6%)	1 (3%)		4 (6%)	0 (0%)		
Calcium channel blockers	Yes	13 (24.1%)	7 (21.2%)	0.349	19 (28.4%)	1 (5%)	0.046	
	No	38 (70.4%)	26 (78.8%)		45 (67.2%)	19 (95%)		
	Not documented	3 (5.6%)	0 (0%)		3 (4.5%)	0 (0%)		
Angiotensin converting enzyme inhibitors	Yes	18 (33.3%)	12 (36.4%)	0.844	23 (34.3%)	7 (35%)	0.531	
	No	33 (61.1%)	20 (60.6%)		40 (59.7%)	13 (65%)		
	Not documented	3 (5.6%)	1 (3%)		4 (6%)	0 (0%)		
Angiotensin receptor blockers	Yes	5 (9.3%)	3 (9.1%)	0.86	7 (10.4%)	1 (5%)	0.382	
	No	46 (85.2%)	29 (87.9%)		56 (83.6%)	19 (95%)		
	Not documented	3 (5.6%)	1 (3%)		4 (6%)	0 (0%)		

**Table 3:** Effects of diagnostic factors on mRS categories at discharge.

		Two category mRS		*P* value	Three category mRS		*P* value
		0-2	>2		0-3	>3	
Stroke etiology	Intracranial arterial stenosis	20 (34.5%)	8 (24.2%)	0.008	23 (32.4%)	5 (25%)	0.294
	Cardioembolism	9 (15.5%)	15 (45.5%)		16 (22.5%)	8 (40%)	
	Others	29 (50%)	10 (30.3%)		32 (45.1%)	7 (35%)	
Occlusion site	Internal carotid artery	12 (34.3%)	3 (14.3%)	0.276	13 (30.2%)	2 (15.4%)	0.814
	Middle cerebral artery (MCA)	14 (40%)	9 (42.9%)		16 (37.2%)	7 (53.8%)	
	Middle carotid artery section M1	1 (2.9%)	3 (14.3%)		3 (7%)	1 (7.7%)	
	Middle carotid artery section M2	3 (8.6%)	4 (19%)		5 (11.6%)	2 (15.4%)	
	Anterior cerebral artery	1 (2.9%)	1 (4.8%)		2 (4.7%)	0 (0%)	
	Posterior carotid artery	4 (11.4%)	1 (4.8%)		4 (9.3%)	1 (7.7%)	
Diagnosis of hypertension at admission	Yes	19 (31.1%)	6 (17.1%)	0.31	20 (27%)	5 (22.7%)	0.853
	No	40 (65.6%)	28 (80%)		52 (70.3%)	16 (72.7%)	
	Not documented	2 (3.3%)	1 (2.9%)		2 (2.7%)	1 (4.5%)	

**Table 4:** Effects of antiplatelet and antihypertensive medications on mRS at discharge.

		Two category mRS scale		*P* value	Three category mRS scale		*P* value
		0-2	>2		0-3	>3	
Aspirin administration within 24-48 hours	Yes	55 (88.7%)	33 (91.7%)	0.463	67 (89.3%)	21 (91.3%)	0.570
	No	7 (11.3%)	3 (8.3%)		8 (10.7%)	2 (8.7%)	
Clopidogrel administration within 24-48 hours	Yes	28 (96.6%)	19 (79.2%)	0.047	34 (91.9%)	13 (81.3%)	0.262
	No	1 (3.4%)	5 (20.8%)		3 (8.1%)	3 (18.8%)	
Clopidogrel with aspirin combination	Yes	23 (63.9%)	20 (69%)	0.667	28 (60.9%)	15 (78.9%)	0.161
	No	13 (36.1%)	9 (31%)		18 (39.1%)	4 (21.1%)	
Labetalol administration	Yes	1 (50%)	2 (40%)	0.809	1 (20%)	2 (100%)	0.063
	No	1 (50%)	3 (60%)		4 (80%)	0 (0%)	

**Table 5:** Binary logistic regression taking mRS score at admission as the dependent variable and sociodemographic factors, past medical, and medication history as independent variables.

mRS two category upon admission	*B*	Sig.	Exp (*B*)	95% C.I. for EXP (*B*)	
				Lower	Upper
Stroke etiology (intracranial arterial stenosis)	1.094	0.099	2.986	0.814	10.962
Previous intake of oral anticoagulant	-1.294	0.063	3.649	0.934	14.247
mRS three category upon admission					
Previous intake of oral anticoagulant	-1.490	0.051	4.437	0.993	19.823
Previous intake of calcium channel blocker	-2.043	0.071	0.130	0.014	1.194

**Table 6:** Linear regression taking mRS score upon hospital admission as the dependent variable and sociodemographic factors, past medical history, and medication history as the independent variables.

Model	Unstandardized coefficients		Standardized coefficients	*t*	Sig.	95.0% confidence interval for *B*	
	*B*	Std. error	Beta			Lower bound	Upper bound
Arrhythmia (positive vs. negative)	0.451	0.247	0.367	1.830	0.078	-0.957	0.054
Diabetes (positive vs. negative)	0.404	0.231	0.285	1.750	0.091	-0.069	0.876
History of percutaneous coronary intervention with stent placement (positive vs. negative)	0.565	0.300	0.388	1.883	0.070	-0.050	1.180
Previous history of calcium channel blocker administration (yes vs. no)	-0.568	0.184	-0.476	-3.086	0.005	0.191	0.945

**Table 7:** Binary logistic regression taking mRS score upon discharge as the dependent variable and sociodemographic factors, past medical history, and medication history as the independent variables.

	Mean	Standard deviation	*P* value	95% C.I. for EXP (*B*)	
				Lower	Upper
Mean difference between mRS at baseline and at discharge	1.73	1.41	<0.001	1.45	2.01
mRS two category upon discharge	*B*	Sig.	Exp (*B*)	95% C.I. for EXP (*B*)	
				Lower	Upper
Stroke etiology		0.017			
Stroke etiology (intracranial arterial stenosis)	1.422	0.035	4.144	1.103	15.574
Stroke etiology (cardioembolism)	-0.330	0.596	0.719	0.212	2.435
ED brain imaging performed 20 minutes after patient presentation to the hospital	1.138	0.037	3.119	1.071	9.080
Hospitalization days	0.1	0.001	1.105	1.104	1.173
mRS three category upon discharge					
Male gender	0.072	0.902	1.074	0.344	3.354
Administration of direct thrombin inhibitors	-0.443	0.782	0.642	0.028	14.715
Administration of antiplatelet clopidogrel	-1.039	0.375	0.354	0.036	3.507
Stroke etiology		0.151			
Stroke etiology (intracranial arterial stenosis)	0.997	0.171	2.711	0.651	11.290
Stroke etiology (cardioembolism)	-0.332	0.645	0.717	0.174	2.956
ED blood glucose more than 180 mg/dL	1.200	0.001	3.301	1.072	1.261
Hospitalization days	0.059	0.01	1.061	1.014	1.109

**Table 8:** Linear regression taking mRS score upon hospital discharge as the dependent variable and the mRS upon hospital admission as the independent variable.

Model	Unstandardized coefficients		Standardized coefficients	*t*	Sig.	95.0% confidence interval for *B*	
	*B*	Std. error	Beta			Lower bound	Upper bound
mRS upon hospital admission	1.252	0.179	0.581	6.991	<0.001	0.896	1.607

## Data Availability

The data used to support the findings of this study are available from the corresponding author upon request.

## Abbreviations

mRS: Modified Rankin Scale
ED: Emergency department
BI: Barthel Index
EQ5D-3L: Health-related quality of life
ICU: Intensive Care Unit
BMI: Body mass index
SPSS: Statistical Package for Social Sciences
SD: Standard deviation
CCB: Calcium channel blocker
CABG: Coronary artery bypass graft
LMWH: Low Molecular weight Heparin
MRI: Magnetic resonance imaging
NIHSS: National Institutes of Health Stroke Scale.
